# Mathematical Model of Propagation of an Aerosol Created by an Impulse Method in Space

**DOI:** 10.3390/ma16165701

**Published:** 2023-08-20

**Authors:** Olga Kudryashova, Sergei Sokolov, Alexander Vorozhtsov

**Affiliations:** 1 Institute for Problems of Chemical and Energy Technologies, Siberian Branch of the Russian Academy of Sciences, St. Socialist, 1, 659322 Biysk, Russia; olgakudr@inbox.ru; 2 Laboratory of Metallurgy Nanotechnologies, National Research Tomsk State University, Lenin Avenue, 36, 634050 Tomsk, Russia; 3 Laboratory for High Energy and Special Materials, National Research Tomsk State University, Lenin Avenue, 36, 634050 Tomsk, Russia; abv1953@mail.ru

**Keywords:** aerosol generation, pulsed generation, particle concentration, gravitational settling, diffusion

## Abstract

When developing neutralization systems for harmful agents, it is necessary to understand the mechanisms of the formation and evolution of an aerosol cloud in a closed or open space. Effective decontamination with aerosol clouds is provided by a rather high particle concentration and dispersion in an open space or on contaminated surfaces. This paper considers neutralization systems based on pulsed powder aerosol generators. It is shown that an aerosol cloud consisting of micron- and submicron-sized particles appears for several seconds after spraying. A further evolution of the aerosol cloud in a room is associated with the gravitational settling, diffusion, and coagulation of particles and their settling on the walls and ceiling. In the case of an open space or a ventilation system in a room, the evolution of the aerosol cloud is affected by the airflow. The main purpose of this paper is to determine the most important parameters and critical conditions of pulsed aerosol generation. A mathematical model is, thus, proposed for pulsed aerosol generation, and its parametric study is conducted in the most typical conditions. The purpose performance predicted by the model is the mass concentration of aerosol particles in the air and on surfaces, depending on the time of particle spraying and dispersion.

## 1. Introduction

Decontaminating aerosols are used to eliminate and remove the adverse effects of environmental hazards, technical aerosol emissions, terrorist attacks, and biological attacks that spread viruses, bacteria, microbes, etc. [1]. Decontamination involves the removal of hazardous and dangerous substances from the atmosphere and surfaces.

The idea of using fine decontaminating aerosols is not new. Artificial fog (liquid-drop fine aerosol) is used in the food industry to reduce the microbial contamination of surfaces [2,3] and in agriculture to improve air quality by reducing airborne dust and microbes [4,5]. Aerosols are created by nebulizers, including ultrasonic ones [6]. Conventional automated spray devices, called no-touch automated disinfection systems, are used to decontaminate large rooms [7].

Most conventional or innovative decontamination techniques have a number of limitations. For example, they require a lot of time for decontamination, cannot remove a sufficient quantity of hazardous substances, or leave traces of their application that are also destructive to health. Traditional methods of spraying disinfectant aerosols often do not allow for achieving high particle dispersion. When talking about spraying powders, then the particles are tens and hundreds of micrometers in size, which means that they quickly precipitate and are not effective. The same applies to spraying liquid particles. An exception is the ultrasonic method, which allows for obtaining particles of 2–4 microns, but this is a very slow method.

Situations requiring a quick response arise as a result of accidents accompanied by emissions of gases or aerosols, terrorist attacks, and the need for the urgent decontamination of premises when dangerous infections are detected. Interest in the study of aerosol propagation in space has increased due to the coronavirus infection [8,9,10,11,12]. In particular, new work emerged simulating the spread of the SARS-CoV-2 virus and similar airborne pathogens in indoor environments [10]. The distribution of the aerosol exhaled by passengers in the cabin of an aircraft [11], in a train compartment [9], and in a classroom [12] were studied. Numerical methods and simulation results certainly provide a lot of insight into the spread of viral aerosols. Meanwhile, an important area of research is the study of methods for neutralizing harmful, including viral, aerosol formations. To accomplish this, the pulse method of spraying can be used.

The proposed technique for decontaminating aerosol clouds utilizes a pulsed generation method, which was introduced in [13,14,15]. This method employs high-energy materials to rapidly spray powders or liquids into the atmosphere. When subjected to a shock wave, powder particles are crushed into smaller particles (less than 10 μm), while liquid drops are reduced in size due to the cavitation mechanism of spraying.

Emergencies that require a fast response can arise from accidents involving gaseous or particulate emissions, terrorist attacks, and urgent indoor air disinfection in the case of contamination. Pulsed aerosol generation allows for the creation of high-speed aerosols and provides the high dispersion ability of particulates. Finer particulates have a larger contact area with hazardous substances, resulting in a more effective decontamination effect with the same mass of the decontaminating agent. The generation process can create an aerosol cloud with a volume of up to 1–20 m^3^, depending on the design that is used and the amount of the dispersed substance.

Aerosol sprays can be solid (powder), liquid, or heterophase (a powder and liquid mix), and the decontamination mechanism depends on the object to be neutralized and the space to be decontaminated, such as closed, semi-closed, or open spaces. Aerosol particles or drops can adsorb harmful particles and gases, react with them, possess catalytic activity, degrade harmful substances, and eliminate/deactivate microbes, among other effects.

In recent research [16], we proposed a mathematical model of the pulsed generation of decontaminating aerosols using high-energy materials (HEMs) with respect to the physicochemical properties of the sprayed material, the HEM properties, and the gas generator parameters. The problems of aerosol propagation in space, aerosol cloud evolution, and settling are also important. Unlike other methods of aerosol generation, these processes are rather specific due to the high dispersion ability of particulates and the cloud generation rate.

The aim of this work is to mathematically model the propagation and settling of pulse-generated aerosol.

## 2. Spraying Process of Primary Aerosol Particles

HEM-based pulse-generated aerosol particles have a size in the range of tens of microns and finer. The aerosol particle size is determined by the powder particle size. Moreover, particle agglomerates disintegrate during spraying, as experimentally and theoretically demonstrated in [14,17], respectively. For such sprayed particles, air resistance becomes of great importance. Air promotes their effective diffusion. Let us repeat the calculation of the primary aerosol cloud given in [17].

The following model is accepted for evaluating the size of the primary aerosol cloud. The explosive HEM allows for instant powder dispersion with the formation of a polydisperse aerosol. In dead air, the aerosol particles spray with an initial velocity of u_p0_ and take the shape of a symmetrical spherical cloud. Let us assume that the initial velocity does not depend on the particle size and is equal to that of the HEM explosion products (sound speed at room temperature), viz., *u_p_*_0_ = 340 m/s.

The equation of motion for a particle with radius *r_p_* takes the form

(1) $$\frac{4}{3}\pi \rho_{p}r_{p}^{3}\frac{du_{p}}{dt}=-\pi r_{p}^{2}C_{D}\frac{\rho u_{p}^{2}}{2}$$

where $\rho_{p}$ is the density of the particle; ρ is the density of the air; *r_p_* is the particle size; *u_p_* is the particle velocity; *C_D_* is the dimensionless factor of resistance, which is calculated by using the Reynolds number Re⁡=2rpρupμ, where *μ* is the dynamic viscosity of air. In this work, the typical Reynolds number is 1<Re⁡<900. In this case, the dimensionless factor of resistance is calculated as [18]

(2) CD=24Re+4Re3.

The integral Equation (1) takes the form

(3) up(t)=36μρprp[(1+6Re02/3)exp(3μρprp2t)−1]−3/2,

where Re0⁡=2rpρup0μ is the Reynolds number at *t* = 0.

The distance *r_c_*_0_ traveled by the particle is determined by the integration of (3):

(4) $$r_{c0}=\int_{0}^{t_{k}}u_{p}\left(t\right)dt.$$

Assume that the upper limit of the integration *t_k_* corresponds to the particle velocity *u_p_*(*t_k_*) = 0.01 *u_p_*_0_. Then, estimate the velocity and distance traveled by particles of different sizes for the following initial parameters: ρ*_p_* = 4.23 × 10^3^ kg/m^3^ (titanium oxide); ρ = 1.205 kg/m^3^; *μ* = 1.81 × 10^−5^ Pa·s; *u_p_*_0_ = 340 m/s.

**Figure 1:**
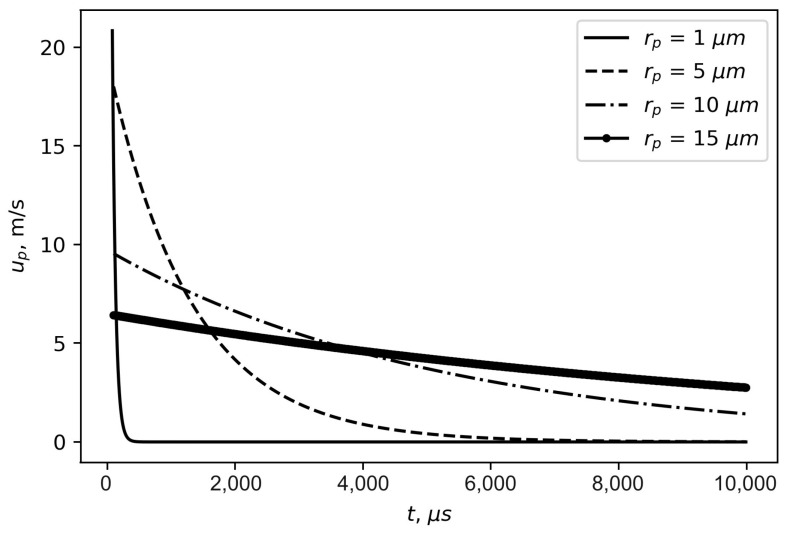
Spray velocity and spray time dependences for four particle sizes.

Table 1 summarizes the values of the particle spray time and the radius of the primary aerosol cloud, depending on the particle radius. The dependences between the spray velocity and spray time for four particle sizes are presented in Figure 1.

As can be seen from these calculations, particle spraying and cloud formation occur for tens of milliseconds, whereas the radius of the primary cloud is not exceeding 5 cm for 20 µm particles.

**Figure 2:**
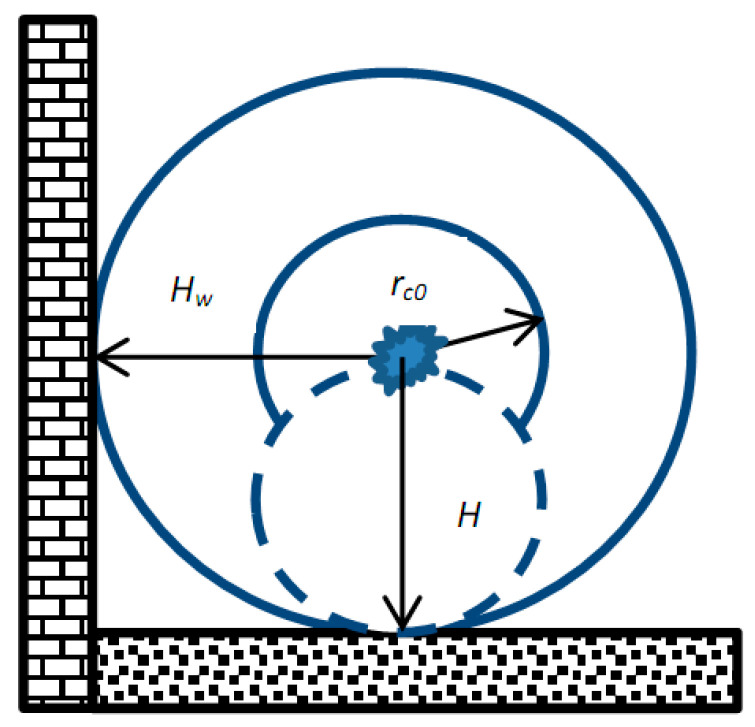
Formation and further propagation of primary aerosol cloud with *r_c_*_0_ radius enabled by particle diffusion or gravitational settling.

Further cloud evolution takes much longer. It is, therefore, advisable to highlight the problem stages. The particle gravitational settling and diffusion are then observed in the space, as illustrated in Figure 2.

Figure 2 schematically illustrates the aerosol spraying from the nozzle of a pulsed generator placed in the center. The primary cloud with radius *r_c_*_0_ gradually expands up to size *H_w_* due to the diffusion or gravitational settling. As reported below, these processes are typical for different particles size and can be separately considered for different powder fractions.

## 3. Fine Aerosol Generation in an Open Space

### 3.1. Gravitational Settling

To calculate the velocity of gravitational settling, let us consider the equation for the stationary settling of spheres according to Stokes’ law at Re < 1:

(5) $$u_{s}=\frac{2\left(\rho_{p}-\rho_{}\right)r_{p}{}^{2}}{9\mu_{}}g,$$

where *g* = 9.81 m/s^2^ is the gravitational acceleration.

The typical time of the particle settling is determined by the following relationship:

(6) $$t_{sk}=\frac{H}{2\times u_{s}},$$

where *H* is the vertical coordinate of the particle.

Table 2 gives the gravitational settling velocity and time of settling from a 1 m height for different particle sizes.

The aerosol cloud is a sphere with volume *V_c_* = 4/3π*r_c_*^3^, where *r_c_* is the cloud radius, which equals the initial radius *r_c_*_0_ determined by (4), regardless of the diffusion expansion. Driven by the gravity force, this cloud moves to the ground at a uniform velocity *u_s_*. Upon arrival at the ground surface (after *t_sk_* time after spraying), the cloud continues to settle at the same velocity. In this case, the cut spherical segment has the volume *V_s_* = π*r*(*t*)^2^(*r_c_* − *r*(*t*)/3), where *r*(*t*) = *u_s_·t*. The volume fraction of the cloud (and the particle mass *m_p_*), which has already settled on the ground surface at time *t*, is calculated as

(7) $$\frac{m_{p}}{m_{p0}}=\frac{V_{s}}{V_{c}}=\frac{3}{4}\frac{r{\left(t\right)}^{2}}{r_{c}^{3}}\left(r_{c}-\frac{r(t)}{3}\right).$$

**Figure 3:**
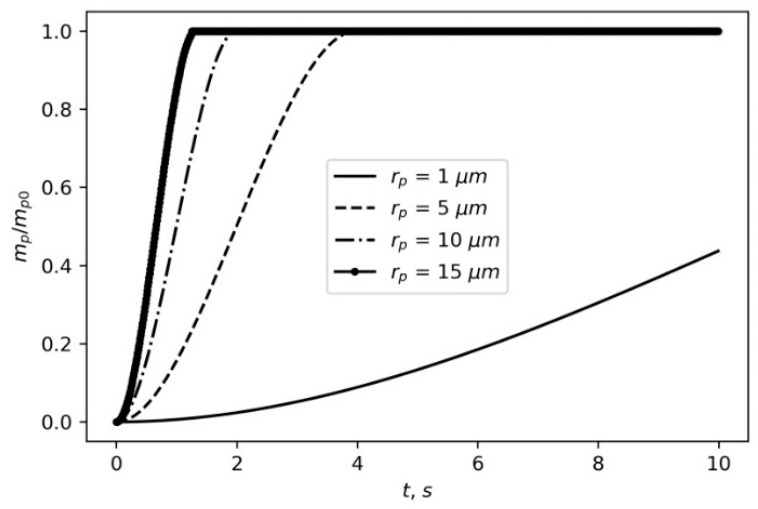
Time dependences of settled aerosol cloud with different particle sizes. The initial cloud radius is calculated from (4).

Figure 3 presents the time dependences of the cloud volume fraction containing particles of different sizes.

It can be seen that the pulse-generated aerosol cloud, consisting of TiO particles with a radius of 10 µm, settles on the ground surface after 1 s of spraying. At the same time, the cloud of TiO particles with a radius of 1 µm settles on the ground after about 1000 s.

By time *t*, the mass fraction of the particles in the air is calculated from

(8) $$1-\frac{m_{p}}{m_{p0}}=1-\frac{3}{4}\frac{r{\left(t\right)}^{2}}{r_{c}^{3}}\left(r_{c}-\frac{r(t)}{3}\right).$$

This relationship can be used to evaluate the possibility of neutralizing harmful agents in the air with regard to the time required and the necessary mass concentration of aerosol.

In practice, it is often important to neutralize harmful agents on surfaces rather than in the air. To estimate the particle concentration on the surface, it is necessary to calculate the surface area on which the particles settle. When the wind does not blow, the surface area is equal to the cross-sectional area of a ball, i.e., π*r_c_*^2^.

When the wind blows at velocity *u_w_*, the surface area covered with particles is $\pi r_{c}^{2}+r_{c}u_{w}t_{s}$, where *t_s_* is the time of the complete cloud settling, i.e., $t_{s}=\frac{2r_{c}}{u_{s}}$. The surface area with settled particles is then found from

(9) $$S_{c}=r_{c}^{2}\left(\pi +\frac{u_{w}}{u_{s}}\right).$$

The higher the wind velocity is relative to the settling velocity, the larger the surface area covered with aerosol particles is. The particle concentration on the surface *m_p_/S_c_* can, however, become insufficient for the neutralization of harmful agents.

#### Gravitational Settling in Convection Conditions

Pulsed aerosol generation should allow for convection and turbulent processes in the air. This is because gaseous emissions—reaction products released from the nozzle of a pulsed generator—contribute to the ambient air, which is initially at rest. Convection currents always occur near the ground that is associated with the temperature difference between the air and surface. Fuks [19] and Davies et al. [20] showed that the following equation is true for cases with convective particle settling:

(10) $$\frac{m_{p}}{m_{p0}}=\frac{V_{s}}{V_{c}}=H\left(1-\exp\left(-\frac{u_{s}t}{H}\right)\right).$$

**Figure 4:**
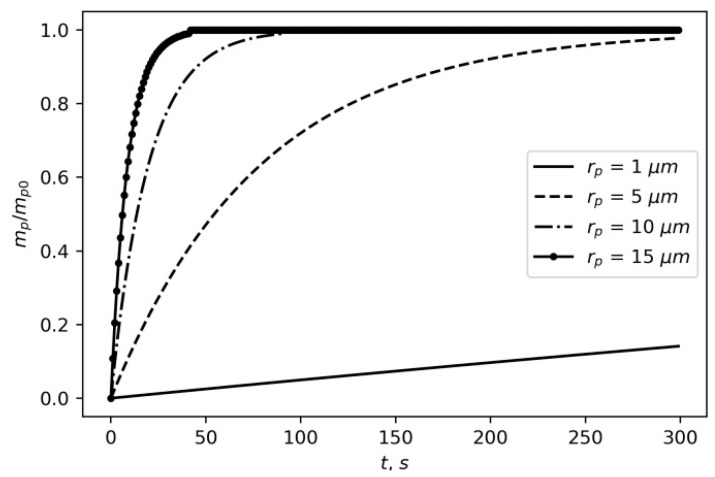
Time dependence of the cloud volume fraction for particles having different radii in convection conditions.

In Figure 4, the volume fraction of the cloud with particles of different radii depends on the time of the gravitational settling with regard to convection and is calculated from (10). As expected, with respect to convection currents, the gravitational settling occurs by an order of magnitude slower.

Let us compare the velocities of the particle gravitational settlement and diffusion in a space.

### 3.2. Particle Diffusion in a Space

In the case of an open space, the particle diffusion is important for the evaluation of the primary cloud expansion during its settling. The experiments carried out in [17] show that the diffusion coefficient for a pulse-generated aerosol is considerably higher than the theoretically calculated Brownian diffusion coefficient. In these experiments, it was 0.0016 m^2^/s, whereas the typical radius of liquid drops is 7.5 µm in the case of a pulse-generated aerosol. This is explained by the fact that an exhaust product jet contributes to the disturbance of dead air, thereby creating turbulent flows promoting particle mixing and accelerated diffusion.

The diffusion coefficient *D* is proportional to the absolute temperature and inversely proportional to the particle radius and air viscosity [21]:

(11) $$D\sim \frac{T}{\mu r_{p}}=\frac{D_{0}}{r_{p}}.$$

The proportional constant *D*_0_ can be identified from the condition *D* (7.5 µm) = *D*_0_/*r_p_* = 0.0016 m^2^/s. It is worth noting that this constant is a free parameter, which must be experimentally clarified.

Based on the problem solution of the suspended particle diffusion, which concentrate in the initial time at a point, the following equation is obtained for the calculation of the average distance $\bar{r}$ traveled by the particle during time *t* [21]:

(12) $$\bar{r}(t)=\sqrt{6Dt}.$$

With respect to the particle diffusion, the aerosol cloud radius increases with time by the value $\bar{r}$:

(13) $$r_{c}(t)=r_{c0}+\bar{r}=r_{c0}+\sqrt{6Dt}.$$

This relation allows us to clarify the particle mass concentration in the air and on the surface during the gravitational settling.

## 4. Fine Aerosol Spray in Closed and Semi-Closed Spaces

The distribution of a finely dispersed aerosol in closed and semi-closed spaces obeys the laws discussed above. But, in addition, it is necessary to consider the settling of particles on the walls (ceiling).

### 4.1. Particle Diffusion and Settling on Walls: Asymptotic Absence of Gravitational Settling

Consider particles that are so small that gravitational settling is neglected. In this case, the particle propagation in space is determined by their diffusion. Let the center of the primary aerosol cloud be located at a distance *H_w_* from the cubic chamber wall. Then, we can obtain the time of arrival at this wall by the cloud boundary as a result of the diffusion:

(14) $$t_{d}=\frac{{\bar{r}}^{2}}{6D}=\frac{{\left(H_{w}-r_{c0}\right)}^{2}}{6D}.$$

**Figure 5:**
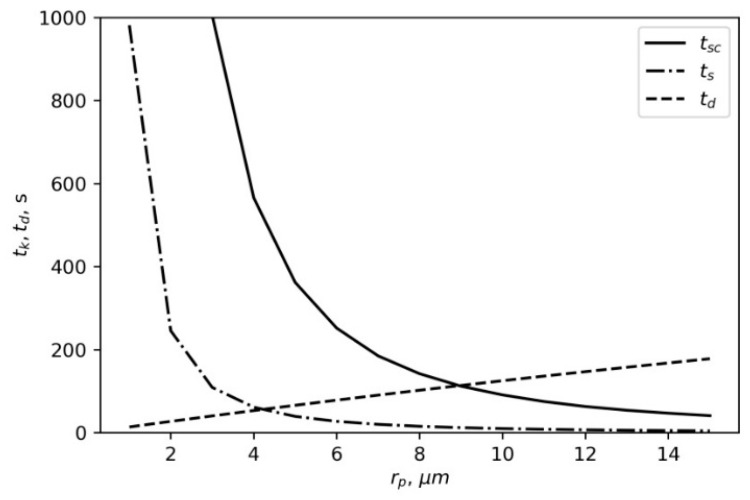
Typical dependences of gravitational settling time *t_s_*, gravitation settling time *t_sc_* in convection conditions, and diffusion time *t_d_* to 1 m distance from the particle radius. Particle density of 4.23 × 10^3^ kg/m^3^ matches the density of TiO particles.

The dependences in Figure 5 show the time of arrival at the wall and the time of settling for different particle sizes.

As presented in Figure 5, TiO particles with a radius of less than 8 or 9 µm are determined by their diffusion, while the latter can be neglected for TiO particles with a radius larger than 9 µm. In this case, the cloud is more likely to settle on the ground than to arrive at the wall and begin to settle on it. Therefore, our further calculations concern the diffusional settling of particles with a radius smaller than 8 µm.

Now, we make the following assumption. Let every particle that arrives at the wall be settled on it; then, after a time, no particles are present in the air. This assumption describes a limiting case of good particle adhesion to the surface. Now, we can find the time of the particle settling on the wall and consider the process dynamics.

By time *t_d_*, the aerosol cloud radius is *r_c_* = *H_w_*, and the particle mass concentration is $C_{m0}=\frac{m_{p0}}{V_{c}}=\frac{3m_{p0}}{4\pi H_{w}^{3}}.$

### 4.2. Particle Settling on a Vertical Wall

The relative velocity of particle settling on a wall is proportional to the particle concentration in the air [19]:

(15) $$\frac{dm_{p}}{dt}=-\beta m_{p}.$$

Coefficient β is proportional to the diffusion coefficient and inversely proportional to the contact area between the cloud and the wall, viz., β *=* β_0_*D*/π*H_w_*^2^. As mentioned above, the effective diffusion coefficient is not equal to the Brownian diffusion coefficient and is higher than in processes of the intensive mixing of airflows.

The solution of (15) is expressible in terms of the exponential function:

(16) $$\frac{m_{p}}{m_{p0}}=1-\exp(-\beta t).$$

**Figure 6:**
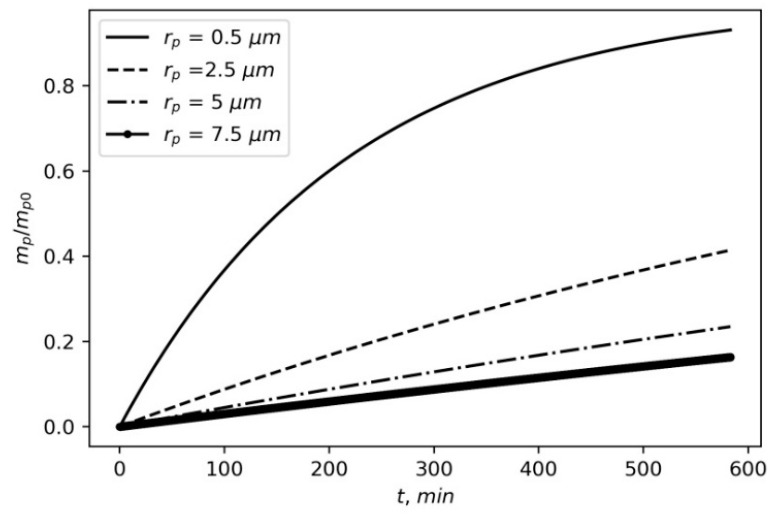
Time dependences between particles with different sizes settled on the wall. The relative mass of the cloud is calculated from (16); *H_w_* = 1 m, β_0_ = 0.01.

Figure 6 presents the time dependence between particles of different sizes settling on the wall. The coefficient β_0_ is selected to correspond to the experimental result obtained in [11] for particles with a radius of 0.5 µm settling on the chamber walls:

#### Particle Settling on Cubic Chamber Walls

Let an aerosol be pulse-sprayed at the center of a cubic chamber with the side 2*H_w_*; then, all the mathematical manipulations above are the same, except for settling, which occurs on six surfaces. The right-hand sides of (15) and (16) must be multiplied by six.

## 5. Air Temperature and Humidity (Preliminary Notes)

According to (11), the higher the air temperature is, the more intense the particle diffusion is. The time taken for particles to reach the wall is inversely proportional to the diffusion coefficient (see (14)) and, consequently, the Kelvin temperature. In contrast, air temperature affects viscosity, which increases when the temperature rises. Hence, the gravitational settling velocity decreases. Thus, at higher air temperatures, particles are more likely to settle on walls and ceilings than on the ground.

Air humidity is not considered in our model. It can be assumed that, in humid air, particulates act as condensation nuclei, and their weight and size grow due to water molecule condensation on their surface. This changes the dynamics of particle settling. Moreover, humid particulates probably adhere better to each other and surfaces and stick together more easily when they collide in the air than dry particles do. Our model assumes that aerosol particles settled on surfaces never leave them. Nevertheless, the model does not consider particle coagulation, i.e., colliding particles do not stick together. These processes are probably affected by air humidity and should be taken into consideration for further model development.

## 6. Conclusions

The paper describes the pulsed generation of decontaminating aerosols using high-energy materials. This spraying technique demonstrates the high velocity of aerosol cloud generation and high particle dispersion compared to other techniques. It is supposed that a pulse-generated aerosol cloud consists of particles capable of decontaminating harmful substances and gaseous emissions.

It was found that the time of aerosol propagation includes primary particle spraying in the order of milliseconds, with the formation of a primary aerosol cloud several centimeters in size, and further cloud evolution, i.e., diffusion expansion, gravitational settling, and settling on surfaces. According to calculations, the particle settling mechanism considerably depends on particle size. Thus, for TiO particles, their critical radius was ~8 µm. Having a larger size, the particles were more likely to settle on the ground than on walls (if the ground and walls were at the same distance from the particles), and vice versa. For a finely dispersed aerosol, the asymptotic case of the absence of gravitational settling is important. In studying the particle settling on both the ground and walls, it was necessary to allow for convection currents, which slowed down the settling process.

The calculated area for the particle settling allowed for detecting the surface concentration. In the case of the wind or ventilation in an open or closed space, the airflow was taken into consideration in (9) to obtain the settling area. Preliminary remarks were made about the effect of the environmental conditions (temperature and air humidity) on the aerosol evolution, which should be taken into account for further model development. The obtained equations for the calculation of the particle mass concentration in the air and on surfaces over time allowed for evaluating the neutralization effectiveness of harmful substances present in space and on surfaces. The free parameters of the proposed model included coefficients *D*_0_ and β_0_. Using the results of experimental work, the proportional constant *D*_0_ was determined as 0.0016 m^2^/s, and β_0_ was selected as 0.01.

## Figures and Tables

**Table 1:** Spray time and primary aerosol cloud radius for four particle sizes.

***r_p_*, µm**	1	2	3	4	5	6	7	8	9	10
***r_c_*_0_, cm**	0.56	1.03	1.53	2.03	2.54	3.04	3.54	4.05	4.55	5.06
***t_k_*, ms**	0.24	0.96	2.16	3.83	5.98	8.61	11.72	15.31	19.38	23.92

**Table 2:** Gravitational settling velocity and time of settling from 1 m height for different particle sizes. Particle density of 4.23 × 10^3^ kg/m^3^ matches the density of titanium oxide.

***r_p_*, µm**	1	2	3	4	5	6	7	8	9	10
***u_s_*, cm/s**	0.05	0.20	0.46	0.81	1.27	1.83	2.49	3.26	4.13	5.09
***t_sk_*, s**	981	245	109	61.3	39.2	27.2	20.0	15.3	12.1	9.81
